# Elimination of inter-domain interactions increases the cleavage fidelity of the restriction endonuclease *Dra*III

**DOI:** 10.1007/s13238-014-0038-z

**Published:** 2014-04-15

**Authors:** Wei Zhuo, Xuhui Lai, Liqing Zhang, Siu-Hong Chan, Fengjuan Li, Zhenyu Zhu, Maojun Yang, Dapeng Sun

**Affiliations:** 1 State Key Laboratory of Biomembrane and Membrane Biotechnology, Tsinghua-Peking Center for Life Sciences, School of Life Sciences, Tsinghua University, Beijing, 100084 China; 2 New England BioLabs, Shanghai R&D Center, Shanghai, 201203 China; 3 New England BioLabs, Inc., Ipswich, MA 01938 USA

**Keywords:** *Dra*III restriction endonuclease, fidelity, substrate specificity, star activity, inter-domain interaction, site-directed mutagenesis

## Abstract

*Dra*III is a type IIP restriction endonucleases (REases) that recognizes and creates a double strand break within the gapped palindromic sequence CAC↑NNN↓GTG of double-stranded DNA (↑ indicates nicking on the bottom strand; ↓ indicates nicking on the top strand). However, wild type *Dra*III shows significant star activity. In this study, it was found that the prominent star site is CAT↑GTT↓GTG, consisting of a star 5′ half (CAT) and a canonical 3′ half (GTG). *Dra*III nicks the 3′ canonical half site at a faster rate than the 5′ star half site, in contrast to the similar rate with the canonical full site. The crystal structure of the *Dra*III protein was solved. It indicated, as supported by mutagenesis, that *Dra*III possesses a ββα-metal HNH active site. The structure revealed extensive intra-molecular interactions between the N-terminal domain and the C-terminal domain containing the HNH active site. Disruptions of these interactions through site-directed mutagenesis drastically increased cleavage fidelity. The understanding of fidelity mechanisms will enable generation of high fidelity REases.

**Electronic supplementary material:**

The online version of this article (doi:10.1007/s13238-014-0038-z) contains supplementary material, which is available to authorized users.

## Introduction

Restriction endonucleases (REases) are components of restriction-modification systems that occur ubiquitously among prokaryotic organisms and are among the basic tools of molecular biology (Roberts, 2005). To date, more than 3600 REases representing more than 250 different specificities have been characterized and classified into four types (Roberts et al., 2003). Type II REases are widely used in genetic technologies because of their stable cleavage pattern and are the most well studied. They recognize short DNA sequences (3–8 bp) and create double strand breaks at constant positions within or close to that sequence to generate new ends with 5′-phosphates and 3′-hydroxyls (Roberts et al., 2003). In particular, type IIP REases recognize palindromic sequences and create symmetrical double strand breaks.

Although REases usually cleave specific DNA sequences accurately, certain REases cleave sequences which are similar, but not identical, to their defined recognition sequences under non-optimal conditions, such as the presence of organic solvent, sub-optimal pH values and high enzyme concentrations. This relaxed specificity has been termed star activity. Star activity has been reported with REases such as *Xba*I, *Sal*I, *Pst*I, *Bam*HI, *Pvu*II and *Eco*RV (Malyguine et al., 1980; Nasri and Thomas, 1987; Robinson and Sligar, 1995). Star activity is not desirable for most REase applications where off-site cleavage is detrimental. Because star activity is relatively weak, the star sites of only a few enzymes have been defined. Interestingly, all identified star sites are only different from the canonical sequence by one base (George and Chirikjian, 1982; Halford et al., 1986; Nasri and Thomas, 1987). Divalent ions and reaction conditions, such as pH, salt concentration and neutral detergents, can enhance or temper the fidelity of some REases (Robinson and Sligar, 1995; Saravanan et al., 2007b). REases of high fidelity have been developed and are commercially available (High Fidelity Restriction Endonucleases, United States Patent No.: US8372619B2, New England Biolabs). The mechanism by which the fidelity increases is still unclear (Wei et al., 2008).

Compared to the fidelity determination mechanism, the catalytic mechanisms of type II REases are well characterized. Several distinct catalytic site motifs and mechanisms have been identified among type II REases (Orlowski and Bujnicki, 2008). The most common catalytic motif, the PD-(D/E)XK superfamily, has evolved to recognize short DNA sequences with the catalytic residues surrounded by a densely packed array of side chains that can fit and interact with the target bases in the major groove (Horton et al., 2004; Orlowski and Bujnicki, 2008). Alternative catalytic motifs, associated with different core protein folds have been experimentally identified in many type II REases. These include the HNH motif in *Kpn*I, *Mnl*I, *Pac*I, *Eco*31I, *Hpy*99I and *Hpy*AV (Chan et al., 2010; Jakubauskas et al., 2007; Kriukiene, 2006; Saravanan et al., 2004; Shen et al., 2010; Sokolowska et al., 2009), the GIY-YIG motif in *Hpy*188I and *Eco*29kI (Ibryashkina et al., 2007; Kaminska et al., 2008), the phospholipase D motif in *Bfi*I (Sapranauskas et al., 2000) and a “half pipe” fold in *Pab*I (Miyazono et al., 2007). A comprehensive bioinformatics study has predicted numerous new members of each of these sub-groups, especially the HNH REases (Orlowski and Bujnicki, 2008).

The conserved structural element of the HNH motif is known as the ββα-metal fold because of its two anti-parallel β-strands connected by a loop of variable length and the α-helix that follows (Mehta et al., 2004). The binding site for a single catalytic divalent metal ion, typically magnesium, is embedded within this fold. To date the co-crystal structures of *Hpy*99I and *Pac*I with their canonical DNA are the only atomic structures that show the intricate protein-DNA interactions of HNH type IIP REases (Shen et al., 2010; Sokolowska et al., 2009). The substrate DNA is severely bent in both structures due to the expansion of the minor groove. In *Pac*I, the DNA distortion is accompanied by unstacked bases and non-canonical A:A and T:T base pairs. This is a clear divergence from the invariant bending of the substrate DNA through the expansion of the major groove by the PD-(D/E)XK REases (Horton et al., 2004).

*Dra*III is a type IIP REase isolated from *Deinococcus radiophilus* ATCC 27603. It recognizes the gapped double-stranded DNA sequence CAC↑NNN↓GTG (↑ indicates nicking on the bottom strand; ↓ indicates nicking on the top strand) and nicks at 5′ of the first G on both strands, thus creating a double strand break with a three base 3′ overhang (Grosskopf et al., 1985). *Dra*III exhibits significant star activity in the presence of Mg^2+^. Considering its star activity and small size (25.7 kDa), we used *Dra*III as a model system to investigate the determinants of cleavage fidelity of REases.

## RESULTS

### Determination of *Dra*III Fidelity Index (FI)

**Figure 1:**
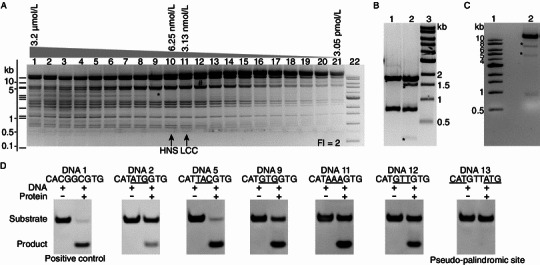
**Determination of*Dra*III Fidelity Index (FI) and star sites**. (A) Determination of *Dra*III Fidelity Index (FI). λ DNA (1.6 nmol/L; 16 nmol/L CACNNNGTG sites) is digested by *Dra*III in a series of two fold dilutions. *Dra*III concentration: Lane 1, 3.2 μmol/L; Lane 10, 6.25 nmol/L; Lane 11, 3.125 nmol/L; Lane 21, 3.05 pmol/L; Lane 22, 1-kb DNA Ladder (NEB). The vertical arrows indicate the two critical points: *HNS*—the Highest REases concentration showing No Star activity and *LCC*—the Lowest REase concentration needed for Complete Cleavage on canonical sites. FI = HNS/LCC, which is 2 in this case. Asterisk represents a star band, and the hash represents a band that resulted from partial cleavage of λ DNA. The theoretical digestion pattern of *Dra*III to λ DNA was predicted using NEBcutter (Vincze et al., 2003) and was shown on the left. (B) *Dra*III star site in pUC19 was predicted to be the CATGTTGTG site. Lane 1: *Bam*HI (cut at nt 417) and *Xmn*I (cut at nt 2298) double digestion on pUC19 generated the 1.9-kb and 0.8-kb bands. Lane 2: *Bam*HI, *Xmn*I and *Dra*III triple digestion on pUC19. Asterisk indicates the star band. According to the approximate size of star bands, the CATGTTGTG site (nt 2033) was hypothesized to be the *Dra*III star site. Cleavage on predicted CATGTTGTG site generated the 1.6-kb and 0.3-kb star bands. Lane 3: 1-kb DNA Ladder. (C) *Dra*III star activity cleaves the CATGTTGTG site in pXba. Lane 1: 1-kb DNA Ladder. Lane 2: pXba was digested by *Dra*III. Asterisk indicates the star bands. *Dra*III star activity generates the expected 6.5-kb and 4.5-kb star bands on pXba. (D) *Dra*III star activity shows selectivity to the central “NNN” part of CATNNNGTG site. There are 11 CATNNNGTG sites in pXba and the sequences containing these sites were tested independently on oligonucleotide duplex DNAs carrying each of the sites (Table S1). The canonical CACGGCGTG site was used as positive control. *Dra*III shows cleavage activity to CATATGGTG, CATTACGTG, CATGTGGTG, CATAAAGTG and CATGTTGTG sites. *Dra*III did not cut the pseudo-palindromic CATGTTATG site

A quantitative definition of star activity is provided by the Fidelity Index (FI), which is the ratio of the highest quantity of a REase showing no star activity during digestion to the lowest quantity needed for complete digestion on canonical sites on a specific substrate DNA (Wei et al., 2008). A higher FI corresponds to a higher cleavage fidelity of the REase. *Dra*III has an FI of 2 on λ DNA under reaction conditions described in MATERIALS AND METHODS (Fig. 1A).

### Determination of *Dra*III star sites

In addition to the expected bands resulting from cleavage of the 10 canonical CACNNNGTG sites, extra discrete bands were observed in the cleavage reactions of λ DNA (Fig. 1A). These bands were attributed to star activity. The defined size of the star bands from cleavage reactions of λ DNA suggests that the *Dra*III star activity is not random and has certain specificity. It is difficult to map the *Dra*III star sites in λ DNA because of its large size (48.5-kb) and the large number of possible star sites. A smaller 2.7-kb pUC19 DNA was used to determine *Dra*III star sites. Although it does not contain the *Dra*III canonical site, the pUC19 DNA was linearized by *Dra*III (data not shown), showing the presence of one or more *Dra*III star sites. Double (*Bam*HI and *Xmn*I) and triple (*Bam*HI, *Xmn*I and *Dra*III) digestion on pUC19 suggest that *Dra*III star activity generates two star bands through one cut (Fig. 1B). According to the approximate size of star bands, the CATGTTGTG site (nt 2033) which only differs from the canonical sequence by one base was hypothesized to be *Dra*III star site. Cleavage on predicted CATGTTGTG star site generated the 1.6-kb and 0.3-kb star bands (Fig. 1B, Lane 2). A larger 22.6-kb plasmid DNA pXba was used to verify the predicted star site. pXba contains three *Dra*III canonical sites (nt 5816, 16551 and 17404 respectively) and one predicted star site CATGTTGTG (nt 21910). It was found that *Dra*III generated the 6.5-kb and 4.5-kb star bands consistent with cleavage at the CATGTTGTG site (Fig. 1C, Lane 2).

Since *Dra*III’s canonical specificity has no preference for the middle three nucleotides (Grosskopf et al., 1985), the specificity of these nucleotides within the star site CATNNNGTG was investigated. There are 11 such sites in pXba and the sequences containing these sites were tested independently on oligonucleotide duplex DNAs carrying each of the sites (Table S1). Among the 11 CATNNNGTG sites (Table S1, DNA2–12), cleavage was observed where NNN = AAA, GTG, TAC, ATG and GTT (Fig. 1D). Apparently the star activity of *Dra*III has a sequence preference for the central three nucleotides, whereas the canonical cleavage activity does not (Grosskopf et al., 1985).

### *Dra*III digests star site sequence in asymmetrical pattern

**Figure 2:**
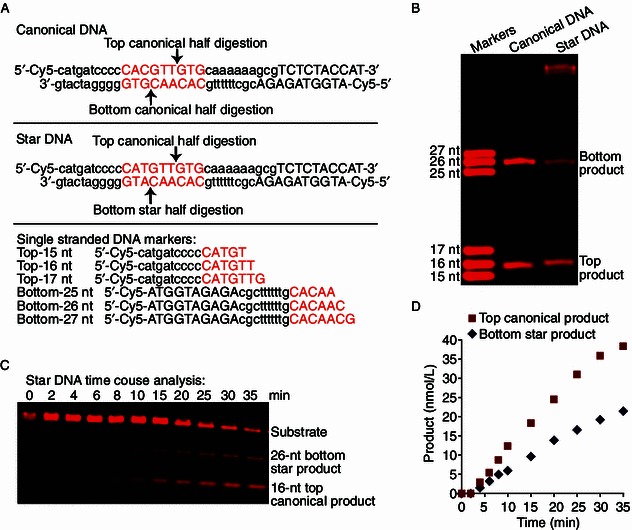
***Dra*III digests star site sequence in asymmetrical pattern**. (A) Sequences of Cy5-labeled DNA substrates. (B) Star activity cleavage occurs in CAT↑GTT↓GTG. Cy5-labeled canonical or star DNA was digested by *Dra*III as described in Supplementary EXPERIMENTAL. The single strand product was separated by TBE urea polyacrylmide gel and compared with synthesized single-stranded markers. Star activity cleavage occurs in CAT↑GTT↓GTG (↑ indicates nicking on the bottom strand; ↓ indicates nicking on the top strand). (C and D) *Dra*III digests a star site sequence in an asymmetrical manner. 100 nmol/L Cy5-labeled star DNA was digested by 500 nmol/L *Dra*III. The samples were collected at the designated time intervals and were analyzed by electrophoresis using TBE urea polyacrylmide gel. The amount of the quantified products was plotted against time

To locate the exact cleavage position within the CATGTTGTG star site, 39-bp duplex DNA which contains canonical or star site and a 5′ Cy5 fluorophore on each strand (Fig. 2A) was digested by *Dra*III and separated by 20% acrylamide urea PAGE (Fig. 2B). The single strand product was compared with synthesized 15, 16, 17, 25, 26 and 27-nt single strand DNA markers (Fig. 2A and 2B). The PAGE result confirmed that *Dra*III star site is CAT↑GTT↓GTG (↑ indicates nicking on the bottom strand; ↓ indicates nicking on the top strand) (Fig. 2B).

To decipher the *Dra*III star activity cleavage mode, 39-bp duplex star DNA was used for time course analysis. Cleavage products were collected at different time points and analyzed by denaturing urea polyacrylamide gel electrophoresis. The 16-nt single strand product (5′-Cy5-catgatccccCATGTT) represents nicking at the top canonical half site and the 26-nt single strand product (5′-Cy5-atggtagagacgcttttttgCACAAC) represents nicking at the bottom star half site (Fig. 2A, star DNA; Fig. 2C). The nicking on the two halves of the *Dra*III star sequence occurs at different rates: the canonical half site GTG was cleaved faster than the star half site CAT (Fig. 2D). No difference in the cleavage rate of the two half sites for the canonical *Dra*III site was observed (data not shown). We further tested whether *Dra*III star activity cleaves the star half site at an equal or slower rate if the star half site CAT is present in both half sites arranged in the pseudo-palindrome (CATGTTATG). *Dra*III showed no significant activity on this pseudo-palindromic site (Fig. 1D, DNA13). This shows that under star conditions *Dra*III cleaves the canonical half site faster than the star half site, and that it requires one canonical half site for cleavage to occur. Altering the flanking sequence (Table S1, DNA14) showed no influence on the cleavage of the CATGTTGTG star site (data not shown).

### Overall protein structure and catalytic sites

**Figure 3:**
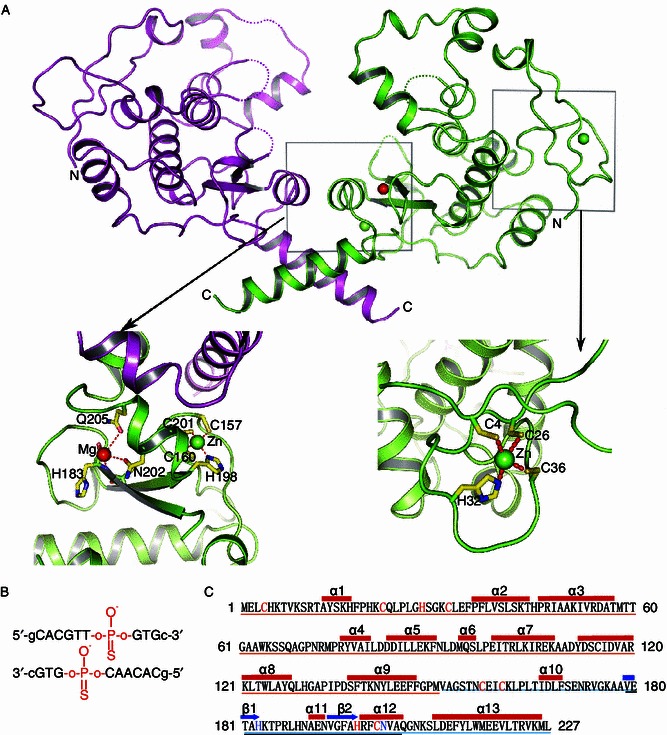
**The*Dra*III overall protein structure**. (A) The complete *Dra*III structure derived in the presence of magnesium chloride. The ion binding motifs are shown. (B) The 11-bp phosphorothioate canonical DNA duplex. (C) The secondary structure of the *Dra*III subunit. Elements of secondary structure are indicated by α-helix (red rectangle) and β-strand (blue arrow). Catalytic residues of the HNH endonuclease motif are indicated with blue font. Zinc-binding cysteine residues are indicated with red font. The location of N-terminal domain, C-terminal domain and ββα-metal motif is indicated by the orange, cyan and black underlines respectively

The structure of *Dra*III was determined in the presence of an 11-bp canonical DNA duplex containing a phosphorothioate at both of the scissile phosphodiester bonds (Fig. 3B). A complete protein structure was derived from a protein-phosphorothioate DNA duplex in the presence of magnesium chloride, although the DNA was not observed in the structure (Fig. 3A and Table 1). *Dra*III exists as a homodimer and dimerization occurs in the C-terminus region where the ββα-metal HNH active site is located. Two zinc- and one magnesium-binding sites are found in each *Dra*III monomer (Fig. 3A).

**Table 1:** Data collection and refinement statistics

	Selenium-methionine protein
**Data collection**	
Diffraction beam	SSRF
Space group	P 2_1_
Unit cell (Å)	*a* = 58.40, *b* = 119.06, *c* = 82.11, *α* = 90, *β* = 92.89, *γ* = 90
Wavelength (Å)	0.979
Resolution (Å)	35.72–2.328 (2.411–2.328)
*R*_sym_ (%)	4.7 (28.9)
*I*/sigma	17.48 (3.05)
Completeness (%)	98.3 (95.5)
Redundancy	2.2 (2.1)
Wilson B factor (Å^2^)	39.3
Number of reflections	47240
**SAD phasing**	
Anomalous scatterers	0.28
Figure-of-merit (FOM)	0.57
FOM after DM	0.66
**Refinement**	
*R*_work_	0.203
*R*_free_	0.255
No. atoms	6782
B factors	
Overall	39.7
Main chain	39.2
Side chain	40.6
Water	35.2
Ligands	29.9
RMSD bond lengths	0.010
RMSD bond angles	1.2
Ramachandran plot statistics (%)	
Favored	97.2
Allowed	2.54
Outliers	0.25
PDB accession codes*	4L0K

Values in parentheses are for the highest resolution shell

*R*_sym_ = Σ_h_Σ_i_|*I*_*h,I*_ − *I*_*h*_|/Σ_h_Σ_i_*I*_*h,i*_, where *I*_*h*_ is the mean intensity of the *i* observations of symmetry related reflections of *h*

*R* = Σ|*F*_*obs*_ − *F*_*calc*_|/Σ*F*_*obs*_, where *F*_*calc*_ is the calculated protein structure factor from the atomic model (*R*_free_ was calculated with 5% of the reflections)

* The protein structure (PDB # 4L0K) have been deposited in the Protein Data Bank (http://www.rcsb.org/)

**Figure 4:**
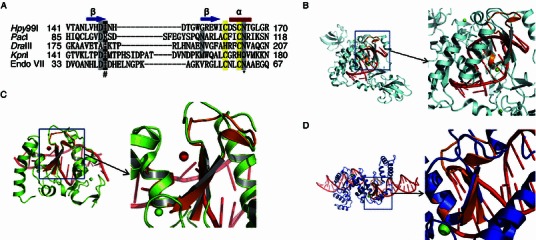
**Comparison of the sequence and structures of HNH REases**. (A) Sequence alignment of the *Dra*III, *Hpy*99I, *Pac*I, T4 Endo VII and *Kpn*I HNH catalytic motif. (B–D) *Dra*III HNH motif (orange) aligned to *Hpy*991 (B: cyan, PDB 3GOX), *Pac*I (C: green, PDB 3M7K) and T4 Endo VII (D: blue, PDB 2QNF), respectively. The zinc ion is shown as green sphere, magnesium ion and DNA are shown in red

The *Dra*III monomer contains a ββα-metal fold (β1-β2-α12) in the C-terminal domain where the HNH active site is located (Fig. 3C). Sequence and structure alignments reveal that amino acid residue N202 in *Dra*III, positioned to coordinate the divalent metal cofactor, corresponds to N113 in *Pac*I, N165 in *Hpy*99I and Q175 in *Kpn*I, among known HNH REases (Fig. 4). H183 in *Dra*III corresponds to H149 in *Hpy*99I and H149 in *Kpn*I, the general base that activates a nucleophile for the hydrolysis of the phosphodiester bond (Fig. 4). To verify their role in catalysis, H183 and N202 were mutated to Ala. Mutants H183A and N202A were overexpressed and were found to be inactive at the cell lysate level (Table 2). Superposition of the *Dra*III closed structure to *Hpy*99I, *Pac*I and T4 Endo VII shows the high structural conservation of the HNH active site (Fig. 4).

**Table 2:** Activity and FIs of *Dra*III variants

*Dra*III variants	Endonuclease activity^a^	FIs^b^
Wild type *Dra*III	+++	=2
**Zinc-binding**		
C4A, C22A, H28A, C32A, C157A, C160A, H198A, C201A	−	N/D
**Catalytic**		
H183A, N202A	−	N/D
**Middle region**		
T181S	+++	=2
T181Y	++	=2
T181A	++++	≥4000
T181G, T181V, T181M, H189A	+++	≥2000
T181N, T181C, T181L	+++	≥1000
T181Q, D55A	+++	≥512
**Mouth region**		
R187A	+++	≥2000
**Hydrophobic patch**		
L129A, L164A	+++	=64
F169A	+++	≥128
V179A	+++	=32

^a^Endonuclease activities of WT *Dra*III and of mutant derivatives were compared at cell lysate level. The cell lysate is diluted by 1, 10, 10^2^ and 10^3^ folds. The “−” means no complete digestion observed in the highest lysate concentration. The “++++” activity means complete digestion observed in 10^3^-fold dilution lane. Because WT shows “+++” activity, the limit of endonuclease activity detectable in this assay corresponded to 10^3^-fold less than or 10-fold higher than WT

^b^The FIs were determined with purified proteins except for T181Y. “N/D” means not detected

A number of HNH endonucleases display a series of histidine and cysteine residues with a characteristic spacing that allows for coordination of a zinc ion in a cross-brace structure. Most HNH endonucleases (REases, homing endonucleases and non-specific endonucleases) contain a CXXC/CXXC zinc-binding domain in the vicinity of the HNH active site that ligates the latter to the rest of the protein. *Hpy*99I and *Pac*I have an extra copy of the CXXC/CXXC domain near the N-terminus (Shen et al., 2010; Sokolowska et al., 2009). The N-terminal zinc binding site (consisting of C4, C22, H28 and C32) and the C-terminal zinc binding site (C157, C160, H198 and C201) of *Dra*III (Fig. 3C) deviate from the CXXC/CXXC configuration. Similar to other HNH REases and homing endonucleases, the two zinc ions are tetrahedrally coordinated by four cysteine/histidine residues in the *Dra*III structure (Fig. 3A).

The coordination of the zinc ion in the second zinc binding site has been shown to play an essential role in the catalytic activity of T4 Endonuclease VII (Giraud-Panis et al., 1995). The removal of the two zinc ions by denaturing the enzyme in urea has also been shown to adversely affect the folding, thermostability and cleavage activity of *Kpn*I (Saravanan et al., 2007a). Alanine mutation of any one of the eight zinc coordinating cysteine or histidine residues rendered the *Dra*III enzyme inactive at the cell lysate level (Table 2).

### Interactions between the N- and C-terminal domains

**Figure 5:**
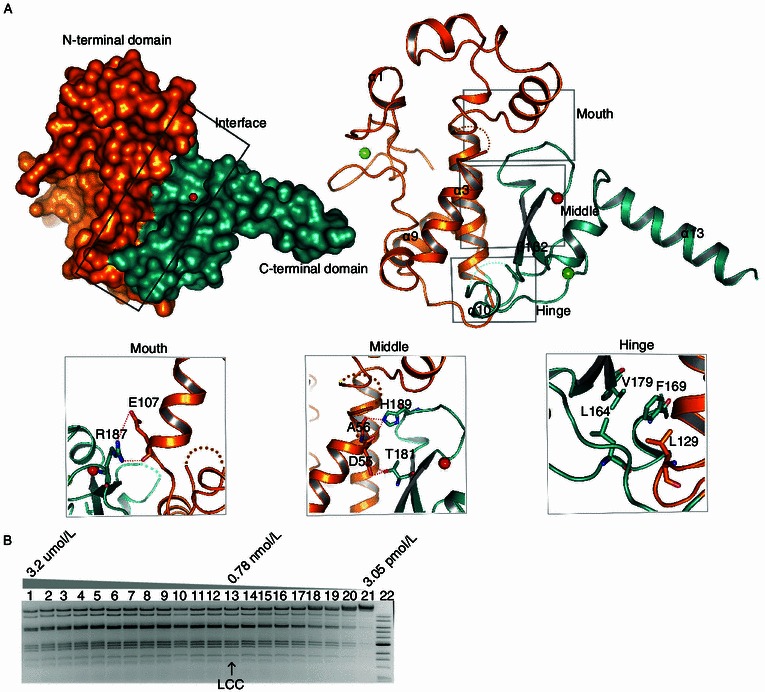
**The interactions between the N-terminal domain and C-terminal domain of*Dra*III subunit**. (A) *Dra*III subunit structure. N-terminal domain in orange; C-terminal domain in cyan; Zinc in green; Magnesium in red. Left: *Dra*III subunit displayed in surface mode. Right: *Dra*III subunit displayed in cartoon mode. Interactions in the mouth, middle region and hydrophobic residues in the hinge region were shown in gray boxes. The locations of potential hydrogen bonds are indicated with red dotted line. (B) Determination FI of *Dra*III T181A. λ DNA was cleaved by diluted T181A. Lane 1: 3.2 μmol/L T181A; Lane 13: 0.78 nmol/L (LCC); Lane 21: 3.05 pmol/L; Lane 22: 1-kb DNA Ladder. FI is larger than 4000. The hash represents a partially digested band. No star band was observed. Disrupting hydrogen bond between T181 and D55 in the middle region remarkably enhanced FI

The *Dra*III structure is composed of two domains: the N-terminal domain (α1–9) and the C-terminal domain (α10–13, β1–2) (Figs. 3C and 5A). The two domains are connected by the turn between α9 and α10, opening up to the solvent at the end of α3 of the N-terminal domain and the turn connecting the two β strands of the ββα-metal fold in C-terminal domain (Fig. 5A, cartoon mode). More importantly, the domain interface opens up to the active site tunnel the substrate DNA would bind to (Fig. 5A, surface mode). Six potential hydrogen bonds are found between the two domains along the domain interface, which can be divided into three regions: the mouth, middle and hinge region (Fig. 5A). A hydrophobic patch is found at the hinge region (Fig. 5A).

To study the role of the potential hydrogen bonds that connect the two domains, a series of mutations were made to the hydrogen bond donors and acceptors along the domain interface. T181 was mutated to Ala to disrupt the two potential hydrogen bonds located at the middle region between T181 and D55 (Fig. 5A, middle region). The mutant T181A showed remarkable high fidelity—no star activity was observed at very high enzyme concentrations (FI ≥ 4000) (Fig. 5B). To verify the role of these hydrogen bonds in cleavage fidelity, mutations T181S and T181Y, which potentially retain the hydrogen bonds, were created. Both mutants T181Y and T181S had the same FI as the WT (Table 2). Mutating T181 further to residues that do not support hydrogen bond formation, namely, Gly, Cys, Val, Met, Leu, Asn or Gln, and D55 to Ala improved the cleavage fidelity to different extents (Table 2). Mutation of T181 to Tyr did not improve the fidelity when tested in cell lysate preparations. Unfortunately, the mutant protein T181Y could not be purified to high enough quantity for further characterization.

Mutation H189A, which potentially disrupts two potential hydrogen bonds in the middle region between H189, D55 and A56 also resulted in high fidelity (Fig. 5A and Table 2). The two potential hydrogen bonds between R187 and E107 are located at the mouth region (Fig. 5A). Mutating R187 to Ala also improved cleavage fidelity (FI ≥ 2000) (Table 2). Mutation of L129, L164, F169, or V179 in the hydrophobic patch at the hinge region to Ala also increased cleavage fidelity, albeit to a lesser extent (Table 2).

### Differential scanning calorimetry (DSC) analysis of the *Dra*III high fidelity mutants

To evaluate the effect of the domain interface mutations on the *Dra*III protein, the melting temperatures (T_m_) of purified *Dra*III WT, T181A, T181G, H189A and R187A proteins were measured by DSC. As shown in Table 3, T181A, T181G and H189A have T_m_ values of 56.7°C, 52.5°C and 56.2°C, respectively, compared to the WT value of 59.1°C. Mutation of T181 and H198 to Ala led to similar and lower T_m_ values supporting the notion that hydrogen bonds were disrupted by each of the mutations. The even lower T_m_ value of the mutant T181G is probably due to the thermodynamic penalty in the folding of the protein caused by the cavity created by the Gly mutation. The correlation between high cleavage fidelity and disruption of hydrogen bonds between the two domains leads us to propose that the high cleavage fidelity is the result of increased conformational flexibility of the mutant proteins. The T_m_ of another high fidelity mutant R187A (T_m_ = 60.7°C^)^ is comparable to that of WT, suggesting that the mutation R187A improves cleavage fidelity through a different mechanism.

**Table 3:** The T_m_ values of *Dra*III variants by differential scanning calorimetry analysis

*Dra*III variants	T_m_ (°C)
Wild type	59.4
T181A	56.7
T181G	52.5
H189A	56.2
R187A	60.7

## DISCUSSION

### *Dra*III is the first HNH REase crystallized which recognizes a gapped sequence

The crystal structures presented here show that *Dra*III contains the ββα-metal fold of the HNH active site. The ββα-metal fold of *Dra*III is highly similar to that of known HNH enzymes such as *Pac*I (Shen et al., 2010) and *Hpy*99I (Sokolowska et al., 2009) (Fig. 4). Structural superposition and sequence alignment show that N202 and H183 of *Dra*III correspond to the magnesium binding residue and the general base, respectively, in *Pac*I, *Hpy*99I and *Kpn*I (Fig. 4). The loss of catalytic activity when these residues were mutated is consistent with this assignment (Table 2). The HNH catalytic motif has been identified in numerous classes of nucleases, from the sequence non-specific DNA colicins (Ko et al., 1999; Pommer et al., 2001), Holliday junction-specific T4 endonuclease VII (Giraud-Panis et al., 1995), to homing endonucleases and REases (Orlowski and Bujnicki, 2008) and most recently in Cas9 of the CRISPR immunity system of bacteria and archaea (Gasiunas et al., 2012; Jinek et al., 2012). Even within the REase family the HNH enzymes exhibit diverse sequence specificity: TTA↑AT↓TAA for *Pac*I, ↑CGWCG↓ for *Hpy*99I, G↑GTAC↓C for *Kpn*I and CAC↑NNN↓GTG for *Dra*III. Among the known HNH REases, the structure presented here is the first for one that recognizes a gapped sequence. The absence of the DNA in the enzyme-DNA co-crystal structure, however, prevented us from examining the protein-DNA interactions through which *Dra*III recognizes the canonical 9-bp sequence with a three base gap in the middle.

### *Dra*III exhibits high star activity with certain sequence specificity

Under standard reaction conditions, *Dra*III has a FI of 2 on λ DNA (Fig. 1A). Our investigation on the *Dra*III star activity revealed a few intriguing properties. First, CATNNNGTG was identified as a strong star site for *Dra*III. Within the context of CATNNNGTG, the *Dra*III star activity has a sequence preference for the central three nucleotides, whereas the canonical cleavage activity does not (Grosskopf et al., 1985). In addition, *Dra*III cleaves the star site in an asymmetrical manner: the canonical half site (GTG) was cleaved faster than the star half site (CAT) (Fig. 2C and 2D), similar to what had been reported with *Eco*RI (Lesser et al., 1990). A substrate containing pseudo-palindromic star half sites CATGTTATG was not cleaved under the same star conditions, showing that at least one copy of the canonical half site is required for double-strand cleavage. It is consistent with the absence of cleavage by *Eco*RI on non-canonical sites containing two non-palindromic base changes (Lesser et al., 1990).

Based on this research and previous studies on cleavage activity on star sites and crystal structures of star-substrate bound REases, a hypothesis is prompted for *Dra*III where a *Dra*III dimer scans through a piece of dsDNA through the 1-D sliding mechanism (Dikic et al., 2012; Jack et al., 1982; Rau and Sidorova, 2010; Wright et al., 1999) and pauses when a canonical half site is recognized by one of the monomers. The presence of a one-base-off half star site and a canonical half site at the correct position triggers the formation of a cleavage competent enzyme-DNA complex where each of the *Dra*III monomer cleaves the bound half site. The star half site is, however, cleaved at a slower rate than the canonical half site due to the thermodynamic penalty to establish non-canonical protein-DNA interactions in an “adaptive conformation” (Lesser et al., 1990; Viadiu and Aggarwal, 2000). As both strands are cleaved the target sequence is destroyed and the enzyme dissociates from the DNA. From this hypothesis, star activity of homodimeric REases is dependent on the propensity of a particular one-base-off star site to form a cleavage competent conformation with the enzyme. The thermodynamic requirements for the formation of such a conformation therefore allows some star sites to be cleaved more effectively than others. According to this hypothesis, REases showing high star activity, such as *Dra*III, may have a lower thermodynamic barrier to adopt the cleavage competent conformation with some star sites. Our results suggest that the sequence of the middle three nucleotides in the context of a star site also contributes to the energy barrier in forming this conformation.

### Eliminating inter-domain interaction improves the fidelity of *Dra*III restriction endonuclease

According to our hypothesis of how star sites are cleaved, one can prevent cleavage at star sites by creating a higher thermodynamic barrier for the enzyme and the bound star substrate to achieve the cleavage competent state, such that favorable interactions between the enzyme and the cognate substrate can generate enough binding energy to overcome the higher thermodynamic barrier, whereas the lack of such interactions between the enzyme and star substrate results in abolition of cleavage.

In this work it was shown that cleavage fidelity of *Dra*III can be improved by mutation of residues involved in the interactions between the two structural domains (Table 2 and Fig. 5). The potential hydrogen bonds between T181 and D55 in the middle region was studied in most detail. Either removing the hydrogen donor or acceptor (mutations T181A, T181G, T181V, T181M, T181C, T181L and D55A) remarkably enhanced cleavage fidelity. Thus, we hypothesize that these interactions, which also make the protein structure less flexible, help to lower the thermodynamic barrier of the *Dra*III-DNA complex to achieve the cleavage competent state. The low thermodynamic barrier allows the *Dra*III-star substrate complex to reach the cleavage competent state with less favorable binding interactions, hence the relatively high star activity of *Dra*III. Disruption of these interactions in the high fidelity mutants on the other hand imposes a higher thermodynamic barrier such that only the binding of the canonical substrate but not the star substrates can generate enough favorable energy to reach the cleavage competent state. This hypothesis is consistent with the higher free energy changes required for the formation of the cleavage-competent transition state for *Eco*RI with star sites than with the canonical site (Lesser et al., 1990), and could potentially be applicable to the star activity of other type IIP REases in general.

It should be pointed out that mutations that reduce the hydrophobicity and steric integrity of the hydrophobic patch located at the hinge region of the interface (L129A, L164A, F169A and V179A) also improved *Dra*III fidelity albeit to lesser extent (Table 2). On the other hand, mutant R187A has a T_m_ value similar to the wild type value (Table 3), suggesting that the hypothesized hydrogen bonds between R187 and E107 are neither present nor important for folding in native *Dra*III. From the *Dra*III structure derived from the protein-DNA co-crystal, R187 potentially interacts with the substrate DNA (Fig. 5A, mouth region). Hence, mutation R187A improves fidelity probably through altering the protein-DNA interaction network. The mechanism of this fidelity improvement will be studied elsewhere.

In this paper, we reported that eliminating inter-domain interaction improves the fidelity of *Dra*III restriction endonuclease. The understanding of fidelity mechanism could be applicable to the star activity of other REases and help to engineer high fidelity REases.

## MATERIALS AND METHODS

### Strain construction, protein expression and purification

The *Dra*III endonuclease gene *draIIIR* was inserted into pAGR3 vector to create the plasmid pAGR3-*draIIIR*. The *Dra*III methyltransferase gene *draIIIM* was inserted into pACYC184 to create the plasmid pACYC184-*draIIIM*. *E. coli* C3081 (NEB) was first transformed by pACYC184-*draIIIM* so that the *Dra*III sites in the host genome could be modified by the constitutively expressed *Dra*III methyltransferase controlled by the *Tet* promoter. The transformed C3081 was further transformed by pAGR3-*draIIIR* to create a stable *Dra*III endonuclease expressing strain. The strain was grown in LB containing ampicillin and chloramphenicol at 37°C. Protein expression was induced by adding IPTG to 0.5 mmol/L and further incubation for 16 h at 37°C. The collected cells were suspended in Buffer A (20 mmol/L potassium phosphate, 100 mmol/L NaCl, 0.1 mmol/L EDTA, 10 mmol/L 2-mercaptoethanol, 5% glycerol, pH7.0) containing 1 mmol/L PMSF before the cells were disrupted by sonication. After centrifugation the supernatant was loaded onto a Heparin HyperD column (Sigma) and eluted with a linear gradient of Buffer B (20 mmol/L potassium phosphate, 1 mol/L NaCl, 0.1 mmol/L EDTA, 10 mmol/L 2-mercaptoethanol, 5% glycerol, pH7.0). Active fractions were eluted at approximately 500 mmol/L NaCl. These fractions were pooled, diluted 5-fold and loaded onto a Source 15S column (GE Healthcare). Protein was eluted with the same NaCl gradient. Active fractions eluted at approximately 300 mmol/L NaCl. Proteins were concentrated and stored. Mutations were constructed by inverse PCR using pAGR3-*draIIIR* as template. The *Dra*III mutants were expressed and purified using the same methods as for wild type (WT).

### Fidelity Index (FI) determination

To measure the FI of *Dra*III and its mutants, a two-fold dilution series of the concentrated protein stock solution was made using Diluent B (300 mmol/L NaCl, 10 mmol/L Tris-Cl, 0.1 mmol/L EDTA, 1 mmol/L dithiothreitol, 500 μg/mL BSA, 50% glycerol; NEB) to give 21 decreasing concentrations of the enzyme (1×, 0.5×, 0.25×, etc.). Two microliters of the diluted enzyme solutions were then mixed with 1 μg λ DNA in a reaction volume of 20 μL in NEBuffer 4 (50 mmol/L potassium acetate, 20 mmol/L Tris-acetate, 10 mmol/L magnesium acetate, 1 mmol/L dithiothreitol, pH 7.9 at 25°C; NEB). The reactions were incubated at 37°C for 1 h and were then quenched by 2 μL of Stop Solution (10×, 200 mmol/L EDTA, 100 mmol/L Tris-Cl, pH 8.0, 0.03% bromophenol blue, 0.94% SDS). The quenched reactions were analyzed by agarose gel electrophoresis. Gel images were obtained using a UV imager (Bio-Rad) on ethidium bromide (EB)-stained gels. The FI was calculated as the ratio of the highest enzyme dilution showing no star activity to the lowest dilution showing complete digestion (Wei et al., 2008). The FIs were determined with purified proteins except for T181Y. The FI of T181Y was determined using cell lysate dilutions.

### DNA cleavage assays

The *Dra*III star sites on pUC19 and pXba were located by incubating the respective plasmid DNAs (1 μg) in 20 μL reactions containing *Bam*HI (20 units), *Xmn*I (20 units) and/or *Dra*III (2 pmol) and 2 μL of 10× NEBuffer 4 at 37°C for 1 h. DNA was analyzed by 0.8% agarose gel electrophoresis followed by EB staining and UV imaging.

The 5′-Cy5 labeled 39-bp DNA duplexes and 15, 16, 17, 25, 26, 27-nt markers were synthesized by Sangon. 100 nmol/L of the canonical or star DNA was incubated with 500 nmol/L of *Dra*III WT in NEBuffer 4 at 37°C for 1 h. The reactions were stopped by adding EDTA to 20 mmol/L. Samples were then analyzed by 20% TBE urea polyacrylamide gel electrophoresis. The 5′-Cy5 labeled 39-bp DNA duplexes were used for time-course experiments. 100 nmol/L of star DNA and 500 nmol/L *Dra*III WT (or 100 nmol/L of canonical DNA and 10 nmol/L *Dra*III WT) were incubated in NEBuffer 4 at 37°C. Samples collected at the designated time intervals were stopped by adding EDTA to 20 mmol/L. The reactions were then analyzed by 15% TBE urea polyacrylamide gel electrophoresis (Bio-Rad). Gel images were obtained using the Typhoon TRIO Variable Mode Imager (GE Healthcare) using 633 nm excitation. The quantification of products was analyzed using the ImageQuant TL software (GE Healthcare).

The 29-bp DNA duplexes were digested by *Dra*III in 20 μL reactions containing 5 μmol/L *Dra*III, 2.5 μmol/L DNA and 2 μL 10× NEBuffer 4 at 37°C for 2 h. The reactions were stopped by adding 2 μL of Stop Solution and analyzed by electrophoresis on 10% non-denaturing TBE polyacrylamide gel. The gels were stained by EB and images obtained by UV imaging.

### Comparison of endonuclease activity of *Dra*III proteins at cell lysate level

*Dra*III mutants were overproduced using the same conditions as the WT enzyme. One milliliter of culture cells was collected and suspended in 150 μL Buffer A and 50 μL glass beads (Sigma). Cells were broken by vortex and cell lysate was collected after centrifugation. The cell lysate was then diluted 1, 10, 10^2^ and 10^3^ folds using Diluent B. Two microliters of the diluted cell lysate solutions were then mixed with 1 μg λ DNA in 20 μL reactions in NEBuffer 4. The reactions were incubated at 37°C for 1 h and then quenched by Stop Solution. The reactions were analyzed by agarose gel electrophoresis.

### Crystallization

*Dra*III and the 11-bp phosphothioate canonical DNA duplex at a 1:1 molar ratio were incubated on ice for one hour and then subjected to size exclusion chromatography on a HiLoad superdex 75 gel filtration column (GE Healthcare) in the presence of 20 mmol/L Tris-Cl, 150 mmol/L NaCl, 5 mmol/L MgCl_2_, 5 mmol/L DTT, pH 7.0. The target *Dra*III and DNA complex was concentrated to 12 mg/mL for crystallization. Crystals were grown at 18°C by the hanging-drop vapor-diffusion method in a reservior solution containing 0.1 mol/L Tris-Cl, 20% 2-propanol, 5% PEG 8000, pH8.0, and then were cryo-protected using a reservoir solution supplemented with 25% (*v*/*v*) glycerol and then flash-frozen in liquid nitrogen. The crystals belonged to space group P2(_1_).

### Structure determination

All the data were collected at the Shanghai Synchrotron Radiation Facility (SSRF) BL17U, and integrated and scaled using the HKL2000 package (Otwinowski and Minor, 1997). Further processing was carried out using programs from the CCP4 suite (Collaborative Computational Project, 1994). Data collection statistics are summarized in Table 1. The real-space constraints were applied to the electron density map in DM (Cowtan, 1994). The final electron density map was of sufficient quality for BUCCANEER (Cowtan, 2006) to be able to build almost the complete model. The final model rebuilding was performed using COOT (Emsley and Cowtan, 2004) and the protein structure was refined with PHENIX (Adams et al., 2002) using NCS and stereochemistry information as restraints. Structural figures were generated in PyMOL (http://www.pymol.org).

### Differential scanning calorimetry (DSC)

Purified proteins were dialyzed into DSC buffer (20 mmol/L potassium phosphate, 200 mmol/L NaCl, 10 mmol/L MgCl_2_, 5% glycerol, pH 7.0) and protein concentration is adjusted to about 6 mg/mL. DSC experiments were performed using a MicroCal VP-Capillary DSC system (GE Healthcare). The experimental scan rate was set at 90°C/h with a temperature range from 10°C to 110°C. The DSC data were analyzed using the Origin 7.1 software and fitted using a two-state model. The Origin analysis reported values for the T_m_ (melting temperature) for each observed transition.

## PROTEIN DATA BANK ACCESSION NUMBERS

The *Dra*III protein structure has been deposited in the Protein Data Bank (http://www.rcsb.org/) with accession number 4L0K.

## Electronic supplementary material

Below is the link to the electronic supplementary material.

Supplementary material 1 (PDF 7 kb)

## Abbreviations

DSC: differential scanning calorimetry
FI: Fidelity Index
REases: restriction endonucleases
T_m_: melting temperature
