# Supplementary material for: Elimination of inter-domain interactions increases the cleavage fidelity of the restriction endonuclease DraIII
Source: Protein Cell. 2014 Apr 15;5(5):357–68. doi: 10.1007/s13238-014-0038-z (PMC3996161; doi:10.1007/s13238-014-0038-z)
Supplement: Supplementary file 1 — Supplementary material 1 (PDF 7 kb) [file 13238_2014_38_MOESM1_ESM.pdf]

Table S1. The 29 bp duplex DNA sequences which contain all 11 CATNNNGTG sites from pXba

Name	Duplex DNA Sequences (5' to 3')	Positions in pXba (nt)	Note
DNA1*	tctctaccatCACGGCGTGgccttccccc	16536-16564	Canonical CACNNNGTG site
DNA2	actgagagtgCACCATATGcggtgtgaaa	170-198	
DNA3	aaagcgaaagCATTAAGTGgctcgctccc	693-721	
DNA4	ttcccagatgCATCCGGTGctgcggcaga	878-906	
DNA5	acttttgcaaCACGTAATGaaataggagt	10063-10091	
DNA6	tgggggcgtaCACTGGATGgcctttgcct	11772-11800	All CATNNNGTG sites in pXba
DNA7	ggcaccagcgCATCTTGTGacgagtcttc	13728-13756	
DNA8	ctcctgtcgcCATCCTGTGaacgccaccg	18080-18108	
DNA9	cgagcttggaCATGTGGTGgttttccata	19422-19450	
DNA10	gtgctcaaccCACACAATGaaaaaattca	19541-19569	
DNA11	gagccggaagCATAAAGTGtaaagcctgg	20398-20426	
DNA12	catgatccccCATGTTGTGcaaaaaagcg	21895-21923	
DNA13	catgatccccCATGTTATGcaaaaaagcg		Pseudo-palindromic CATGTTATG site
DNA14#	caggttcgggCATGTTGTGttttaaggag		Flanking sequence control

*DNA1 which contains DraIII canonical CACNNNGTG site from pXba is a positive control. #DNA14 is a flanking sequence control which shares the same central CATGTTGTG site in DNA12 but different flanking sequences.
